# A novel totivirus and piscine reovirus (PRV) in Atlantic salmon (*Salmo salar*) with cardiomyopathy syndrome (CMS)

**DOI:** 10.1186/1743-422X-7-309

**Published:** 2010-11-10

**Authors:** Marie Løvoll, Jannicke Wiik-Nielsen, Søren Grove, Christer R Wiik-Nielsen, Anja B Kristoffersen, Randi Faller, Trygve Poppe, Joonil Jung, Chandra S Pedamallu, Alexander J Nederbragt, Matthew Meyerson, Espen Rimstad, Torstein Tengs

**Affiliations:** 1 Section for immunoprophylaxis, National Veterinary Institute, P.O. Box 750 Sentrum, 0106 Oslo, Norway; 2 Section for fish health, National Veterinary Institute, P.O. Box 750 Sentrum, 0106 Oslo, Norway; 3 Section for epidemiology, National Veterinary Institute, P.O. Box 750 Sentrum, 0106 Oslo, Norway; 4 Department of basic sciences and aquatic medicine, Norwegian School of Veterinary Science, P.O. Box 8146 Dep, 0033 Oslo, Norway; 5 Broad Institute of MIT and Harvard, Cambridge, MA 02142, USA; 6 Centre for Ecological and Evolutionary Synthesis (CEES), University of Oslo, 0316 Oslo, Norway; 7 Department of Medical Oncology, Dana-Farber Cancer Institute, Harvard Medical School, Boston, MA 02115, USA; 8 Department of food safety and infection biology, Norwegian School of Veterinary Science, P.O. Box 8146 Dep, 0033 Oslo, Norway; 9 Section for virology and serology, National Veterinary Institute, P.O. Box 750 Sentrum, 0106 Oslo, Norway

## Abstract

**Background:**

Cardiomyopathy syndrome (CMS) is a severe disease affecting large farmed Atlantic salmon. Mortality often appears without prior clinical signs, typically shortly prior to slaughter. We recently reported the finding and the complete genomic sequence of a novel piscine reovirus (PRV), which is associated with another cardiac disease in Atlantic salmon; heart and skeletal muscle inflammation (HSMI). In the present work we have studied whether PRV or other infectious agents may be involved in the etiology of CMS.

**Results:**

Using high throughput sequencing on heart samples from natural outbreaks of CMS and from fish experimentally challenged with material from fish diagnosed with CMS a high number of sequence reads identical to the PRV genome were identified. In addition, a sequence contig from a novel totivirus could also be constructed. Using RT-qPCR, levels of PRV in tissue samples were quantified and the totivirus was detected in all samples tested from CMS fish but not in controls. *In situ *hybridization supported this pattern indicating a possible association between CMS and the novel piscine totivirus.

**Conclusions:**

Although causality for CMS in Atlantic salmon could not be proven for either of the two viruses, our results are compatible with a hypothesis where, in the experimental challenge studied, PRV behaves as an opportunist whereas the totivirus might be more directly linked with the development of CMS.

## Introduction

Cardiomyopathy syndrome (CMS) is a severe disease primarily affecting large farmed Atlantic salmon (*Salmo salar *L.). It was first reported in farmed salmon in Norway in the mid-1980s [1,2], but has also been documented in farmed salmon in the Faroe Islands [3,4], Scotland [5] and Canada [6]. In 2003, CMS-like lesions were also reported in wild Atlantic salmon in Norway [7]. Classically, the disease appears without prior clinical signs in 2 to 5 kg sized fish from 12 to 15 months after transfer to sea water until slaughter and may cause substantial economic losses [5,8]. The histopathological changes of CMS are characterized by moderate to severe inflammation of the heart, dominated by mononuclear cell infiltration, mostly limited to the endocardium and spongy myocardium in the atrium and ventricle [9,10]. Pathological changes in the compact myocardium and epicarditis are rare and not considered typical findings.

Heart and skeletal muscle inflammation (HSMI) is another disease of farmed Atlantic salmon in which cardiac lesions are prominent, and is considered a differential diagnosis to CMS. HSMI has emerged as a disease entity in Norwegian salmon farming with increasing importance in the last decade. The disease outbreaks are, in contrast to CMS, usually reported in smaller fish (0.3 to 1 kg) 5 to 9 months after transfer to sea water [11]. The morbidity, as estimated by histopathology, may be high in affected cages, but the accumulated mortality generally stays below 20%. The histopathological changes of HSMI are characterized by moderate to severe myocarditis with inflammation-associated necrosis of both spongy and compact myocardium in the ventricle. Other consistent findings are moderate to severe epi- and endocarditis [12,13]. In contrast to fish with CMS, fish with HSMI seem to be able to recover with time.

We recently reported the finding and the complete genomic sequence of a novel reovirus; piscine reovirus (PRV), which is associated with HSMI in Atlantic salmon [14]. In the present work we have studied the potential role of PRV and other infectious agents in the development of CMS using high throughput sequencing, PCR and *in situ *hybridization. Our results indicated that a hitherto unknown totivirus might be relevant for the development of CMS whereas PRV may not be causally associated with the disease.

## Materials and methods

### Pyrosequencing and PRV quantification in field samples

Total RNA was extracted using the RNeasy Lipid Kit (QIAGEN AB, Oslo, Norway), from three specimens: two heart samples from fish from a CMS outbreak (peak phase with high mortality in the population) and one combined heart and head kidney sample from an experimentally challenged fish (nine weeks post inoculation; see details below and [10]). The RNA was DNase treated using TURBO DNA-free (Applied Biosystems/Ambion, Austin, TX, USA) and reverse transcribed/amplified using the QuantiTect kit (QIAGEN AB) according to manufacturer's instructions. Approximately 1 μg of RNA was used as template in each multiple displacement amplification (MDA), which was allowed to proceed for two hours. cDNA from the three reactions was combined in equal amounts and a library with a Multiplex Identifier tag was prepared according to the GS FLX Titanium General Library Preparation Method Manual (454 Life Sciences, a Roche company, Branford, CT, USA). The library was titrated and amplified using the large volume emulsion preparation protocol and sequenced using a Genome Sequencer FLX instrument and GS FLX Titanium chemistry (454 Life Sciences). Sequencing was done by the The Norwegian High-Throughput Sequencing Centre (NSC) at the University of Oslo.

### Virus PCR

Reverse transcription quantitative PCR (RT-qPCR) for quantification of viral PRV RNA in CMS field samples was performed as described earlier [14] using the minor groove binding (MGB) assay targeting the L1 genomic fragment. PRV RNA in head kidney and heart from 21 fish from four outbreaks of CMS was quantified and compared to assumed healthy farmed fish.

Samples from challenged fish and controls were obtained from 'Challenge group 1' described in a previous publication [10]. Briefly, 100 unvaccinated wild strain Atlantic salmon were intraperitoneally (i.p.) injected with 200 μl supernatant of tissue homogenate mix from six CMS diagnosed fish. Samples of heart, head kidney, spleen and liver were taken from 5 fish every third week 0 to 36 wpc (weeks post challenge), and directly frozen at -80°C. Total RNA was isolated using the RNeasy Mini Kit (QIAGEN AB). Each PRV RT-qPCR run contained 100 ng RNA in a volume of 12.5 μl and the PCR protocol for detection of PRV described in Palacios et al. [14] was used. Head kidney samples were also screened for the presence of IPNV (Infectious Pancreatic Necrosis Virus), SPDV (Salmon Pancreas Disease Virus) and nodavirus by RT-qPCR. The fish cultivation and research facilities were located in an area geographically separated from the endemic area of CMS in Norway.

A real-time PCR assay was designed for the totivirus found in the 454 sequence database. A protocol identical to the one described above was used with forward primer TTCCAAACAATTCGAGAAGCG, reverse primer ACCTGCCATTTTCCCCTCTT and the MGB probe CCGGGTAAAGTATTTGCGTC (all written in 5'-3' direction; Applied Biosystems, Life Technologies Corporation, Carlsbad, California, USA). The assay was used qualitatively to test both a panel of CMS outbreaks, control samples and fish from the experimental transmission.

### *In situ *hybridization

*In situ *hybridizations were performed on heart sections as described earlier [14] using LNA probes (Exiqon, Vedbaek, Denmark) targeting PRV genomic segment L2 [14] and the totivirus RNA-dependent RNA polymerase gene (probe sequences CTTCCGCTACCATCCTGAGATA and TTTCTTCGACACACCTTTCCGC; 5'-3' direction). Viral RNA was visualized using aminoethylcarbazole (AEC). Heart sections of healthy fish served as controls.

### Comparison of PRV from CMS- and HSMI-affected fish

Genetic variation between PRV sequences from CMS and HSMI outbreaks was estimated by comparing the obtained CMS PRV reads with the corresponding consensus genome segments from HSMI PRV (GenBank accession numbers GU994013-GU994022). Using the CLC Main Workbench (CLC bio, Aarhus, Denmark) and default settings, each of the known HSMI PRV genome segments were consecutively used as a reference for *in silico *assemblies of the CMS PRV reads. Each CMS PRV assembly was inspected manually to identify and evaluate conflicts between individual CMS PRV reads and the HSMI PRV reference genome segments.

## Results

### High throughput sequencing

The pyrosequencing gave a total of 556,947 reads with average length of 240 bases. Data are archived at the National Center for Biotechnology Information (NCBI) Sequence Read Archive (SRA) under accession SRP002117. In order to perform computational subtraction of the reads, removal of ribosomal RNA reads or assembly of read contigs would normally be performed, but due to the possibility of having a significant number of chimerical reads (the QuantiTect kit includes a ligation step were the reversely transcribed cDNA fragments are ligated to facilitate MDA) such filtering of results was not done. Instead, all reads were analyzed using Mega BLAST and a database comprising all nucleotide sequences annotated as viral in the nr database at NCBI was compiled. Mega BLAST searches were conducted using word size 28 and for each read the match that gave the highest bit score was recorded. Results were tabulated and showed a large number of hits to a relatively small number of entries in our viral sequence database. Many of the sequences that showed a high degree of similarity to our reads were obvious errors in the nr database (incorrectly annotated/assembled sequences, vector sequences etc.) and could be discarded. Only four groups of sequences gave credible matches; eight reads were (imperfect matches) to Chum salmon reovirus (accession number AF418297; [15]), two matched Atlantic salmon swim bladder sarcoma virus (acc. no. DQ174103; [16]) and 1,084 reads matched the PRV genome with a very high degree of sequence similarity. A 2,914 basepair sequence contig comprising approximately 2,600 reads from what appeared to be a novel virus could also be constructed. Alignment of conserved amino acid sequence motifs in the RNA-dependent RNA polymerase (RdRP) of the *Totiviridae *indicated that the virus was related to this virus family [17]. Using protein BLAST with default parameters and 290 amino acids of *RdRP*, the closest match was a gag-pol protein (27% identity; 45% positive) from a totivirus infecting *Giardia lamblia *[18]. Partial *RdRP *was control-sequenced using Sanger chemistry and used as target for an MGB RT-qPCR assay and for *in situ *hybridization (GenBank accession number HQ401057).

PRV reads were assembled using the software Sequencher (version 4.5; Gene Codes Corporation Ann Arbor, MI, USA) and revealed approximately 85% coverage of the PRV genome. This was further increased to 94% coverage using Sanger sequencing of PCR amplified fragments (data not shown). The genomic segments showed considerable variation in coverage, both regarding the relative part of the segments covered by reads and the depth of coverage (Table 1). In particular, segments S1 and S2 showed relatively poor coverage. Most of the longer fragments had reads that indicated the presence of missense mutations.

**Table 1:** Estimation of genetic variation between HSMI PRV genome segments and CMS PRV sequence reads.

Segment	Length (nt)	Transversions			Transitions			Amino acid changes			**Coverage**^1^	**Depth**^2^
		100%^3^	>50%^4^	≥2^5^	100%	>50%	≥2	100%	>50%	≥2	-	-

**L1**	3916	0	3	8	6	31	72	2	1	9	88.6%	13.55

**L2**	3935	0	5	5	1	60^6^	44^7^	0	4	11	96.3%	20.02

**L3**	3911	0	0	3	0	3	17	0	0	0	85.3%	17.43

**M1**	2383	0	1	0	13	0	3	0	0	0	63.7%	6.45

**M2**	2179	0	0	2	0	0	3	0	0	4	77.6%	28.92

**M3**	2403	0	0	0	1	0	6	0	0	2	84.7%	8.03

**S1**	1040	0	0	0	3	0	0	0	0	0	34.2%	3.15

**S2**	1329	0	0	0	0	3	0	0	0	0	33.1%	3.02

**S3**	1143	0	0	0	2	5	1	0	0	0	46.5%	3.00

**S4**	1081	0	0	0	0	0	0	0	0	0	54.0%	7.97

1 - Relative part of HSMI PRV segment covered by at least two reads in the CMS PRV sequence dataset.

2 - Average number of times each nucleotide position was sequenced (full length fragment).

3 - All CMS PRV reads exhibited the indicated feature.

4 - The majority of the CMS PRV reads exhibited the indicated feature.

5 - At least two CMS PRV reads exhibited the indicated feature.

6 - The majority of these transitions were found in the 5'-half of L2.

7 - The majority of these transitions were found in the 3'-half of L2.

### PRV PCR

**Figure 1:**
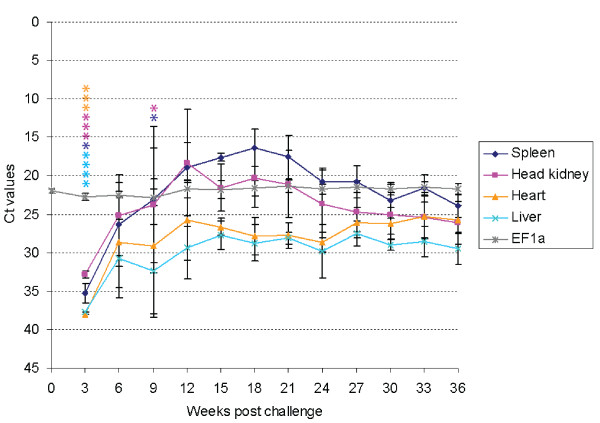
**Quantification of PRV RNA ± standard deviation (Ct values) in heart, spleen, head kidney and liver after intraperitoneal injection challenge using RT-qPCR (n = 5/time point)**. Quantification of EF1α in heart tissue is shown as control. Asterisks indicate negative PCRs.

Screening of samples from field outbreaks of CMS revealed only a modest increase in viral loads compared to the elevated PRV titers seen in HSMI fish (Additional File 1, Table S1 and [14]). In order to investigate further the potential role of PRV in the experimental challenge we quantified the viral loads in various organs throughout the experiment. A general increase with time in PRV RNA levels in all organ samples was found by RT-qPCR 0 to 12 wpc (Figure 1). Six wpc, all organ samples of all analyzed fish were positive. The levels of PRV RNA peaked in head kidney 12 wpc (Ct 18.2) followed by spleen 18 wpc (Ct 16.3). In heart and liver, the PRV levels were in general lower than in the spleen and head kidney, and remained stable from 15 wpc and onwards with the highest levels detected 33 wpc (Ct 25.2) and 15 wpc (Ct 27.7), respectively. Heart control samples taken 3, 6 and 9 wpc (*n *= 2/time point) were PRV negative and the fish population was negative for SPDV and nodavirus. Low and steady levels of IPNV (37 positives of 67 samples, mean Ct ~30) were detected in the head kidney from 6 wpc and onwards.

**Table 2:** Piscine totivirus PCR results.

Sample set	Type of sample	Tissue	Pos/*n*
440	CMS outbreak	Heart/kidney	4/4
479	CMS outbreak	Kidney	5/5
66	CMS outbreak	Heart/kidney	5/5
89	CMS outbreak	Heart/kidney	4/4
130	CMS outbreak	Heart/kidney	10/10
52	CMS outbreak	Heart/kidney	4/4
TP	CMS outbreak	Heart	10/10
253	HSMI outbreak	Heart/kidney	0/2
694	HSMI outbreak	Heart/kidney	0/2
SK07	HSMI outbreak	Heart/kidney	0/2
PD561	Healthy, farmed Atlantic salmon	Heart/kidney	0/6
PD1911	Healthy, farmed Atlantic salmon	Heart/kidney	0/6
PD	Healthy, farmed Atlantic salmon	Heart/kidney	0/10
IRL	Healthy, farmed Atlantic salmon	Heart	0/3
WH	Healthy, wild Atlantic salmon*	Heart	0/23
Wpc0	CMS experimental transmission, prior to challenge	Heart	0/5
Wpc3	CMS experimental transmission, week 3	Heart	1/2
Wpc6	CMS experimental transmission, week 6	Heart	2/2
Wpc9	CMS experimental transmission, week 9	Heart	2/2
Wpc21	CMS experimental transmission, week 21	Heart	5/5
Wpc33	CMS experimental transmission, week 33	Heart	4/4
Wpc36	CMS experimental transmission, week 36	Heart	4/4

* - multiple locations along the Norwegian coast.

### Totivirus PCR

Testing of samples from field outbreaks of CMS showed a perfect correlation between the presence of totivirus RNA and CMS (Table 2). All the control groups, including HSMI outbreaks, were negative. Based on the observation that there appeared to be a consistent relationship between the presence of virus and disease, results were scored qualitatively (Table 2). Control fish from the experimental challenge were negative and by week six post challenge, all fish tested were positive for the virus (Table 2).

### Histopathology and localization of virus

**Figure 2:**
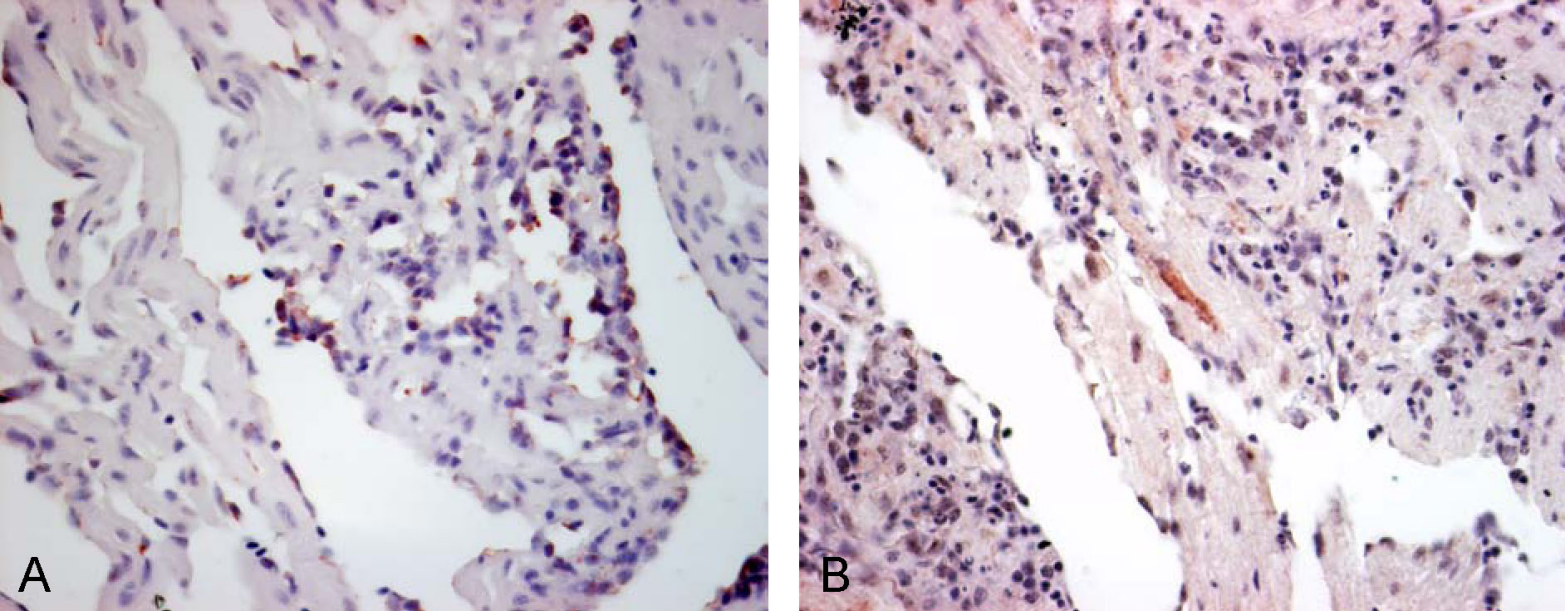
***In situ *detection of PRV RNA and totivirus RNA (red) in heart sections from CMS diagnosed fish**. (A) PRV RNA, (B) totivirus RNA. Sections were counterstained with Meyer's hematoxylin (blue).

The histopathological changes in the transmission experiment have previously been described in detail [10]. Briefly, the severity of atrial lesions peaked at 12 wpc, and remained at this level throughout the study. Ventricular spongy layer lesions were found in ~50% of the challenged fish from 12 wpc and onwards, and epicardial changes were mild and decreased towards the end of the study. Staining for PRV RNA by *in situ *hybridization was seen in the spongious as well as the compact myocardium, and in endocardial cells of areas with distinct lesions and cellular infiltration. Staining for totivirus RNA was seen in myocardial cells associated with characteristic lesions in the spongious myocardium (Figure 2). Control sections were negative.

## Discussion

The newly described piscine reovirus (PRV) belongs to the Orthoreovirus/Aquareovirus group [19] within the *Reoviridae *family [14]. Viruses of the two genera can infect large groups of animals and cause diseases with a broad range of manifestations; from inapparent to lethal. The reoviruses are often considered 'orphan' as they have not been definitely linked to particular diseases. PRV is widely distributed in farmed Atlantic salmon in sea water, and can be found in healthy as well as in diseased fish [14]. It is thus not surprising that the virus was present in the inoculum used in our CMS experimental challenge. Totiviruses, on the other hand, are primarily known to infect unicellular organisms such as *Saccharomyces cerevisiae *and protozoans like *Leishmania *and *Giardia *spp. [20], but the host range has recently been found to include penaeid shrimp, insects and some totiviruses can also be grown in mammalian cell cultures [21]. Totiviruses are naked dsRNA viruses and to our knowledge the closest link to our observation of a totivirus in fish is the penaeid shrimp infectious myonecrosis virus (PsIMNV) which causes necrosis in the skeletal muscle of shrimp [22].

In the present study, histopathological lesions in the heart consistent with CMS were observed from week 6 to the end (36 weeks) of the experimental infection. The PRV and totivirus infections appeared to be persistent, not only in immunologically important organs such as the head kidney and spleen that often harbor persistent and covert viral infections, but also in the heart, the organ where the major histopathological lesions of CMS are found. Neither HSMI nor CMS have been found in the fresh water phase of farmed Atlantic salmon, but both diseases occur after transfer to sea water. HSMI is normally observed 5 to 9 months after transfer while clinical CMS first appears after 12 to 18 months. Independent challenge trials, as well as epidemiological studies, have indicated that CMS has an infectious cause [9,10]. In combination with the late onset of CMS relative to sea water transfer this indicates that a persistent infection is involved in the etiology. If an acute infection is the primary cause of the disease, it should typically have been observed also at earlier stages after sea water transfer. In the field, CMS is often observed as sudden death of large fish, caused by rupture of the atrium or sinus venosus and resultant cardiac tamponade [2]. However, lesions characteristic of CMS develop over several months until the myocardial lesions are severe and widespread and the fish can no longer compensate for the compromised function. Both experimental infections and field observations strongly indicate that CMS is a chronic disease, culminating in sudden death, with moderately elevated mortality rates. In cultured shrimp, the totivirus PsIMNV causes a persistent, slowly progressive disease appearing as extensive necrotic areas in striated muscles, with presence of the virus in heart muscle [23]; a disease progression which has some resemblance to CMS of farmed salmon, characterized by degeneration and necrosis of the inner, spongious myocardium of the ventricle and the atrium [2].

Significantly lower levels of PRV were found in fish from field outbreaks of CMS than fish with a HSMI diagnosis. The totivirus gave a more compelling result where a perfect correlation was found between presence of the virus and disease. Additional non-infectious factors such as fast growth have been suggested to be important for development of CMS, and the highest mortality is usually seen among fish with the highest condition factor. Other contributing factors may include lack of exercise, nutritional factors, environmental factors, stress and co-infections with other known or unknown pathogens able to induce persistent infection with heart involvement. It has been proposed that CMS may be linked to previous viral infections, and an epidemiological link between IPNV outbreaks in early sea water phase and later outbreaks of CMS has been suggested [8,24], but experimental transmission of CMS has been demonstrated in the absence of IPNV [9].

Our sequence data were derived from total RNA extracted from salmon sampled during field outbreaks of CMS and fish where CMS had been induced through experimental transmission. Viral reads were identified using nucleic/amino acid sequence similarity search tools and thus the absence of other candidates becomes significant. The possibility that another, causal virus, was transferred along with PRV and the totivirus in the experimental challenge cannot be excluded. Bioinformatics tools may not have identified the virus, or viral titers may have been too low for detection.

For PRV to be causally involved in two distinct diseases such as CMS and HSMI, the most likely explanation would be that there are different genotypes of the virus. The fish farming industry has many similarities with the poultry industry, such as the production of a large number of a single animal species in an industrial scale within a confined area. The avian reoviruses (ARV) of the Orthoreovirus genus are important pathogens of poultry and cause considerable economic loss in the industry. Like PRV, ARV is ubiquitous in production units and the etiology of specific diseases attributed to ARVs are difficult to reproduce experimentally [25]. The pathogenicity of ARV strains differs considerably and the infections may not cause recognizable diseases or clinical signs at all. The gene encoding the neutralizing antibody inducing viral attachment protein σC, shows nucleotide variation in circulating field strains that has affected the efficacy of vaccines [26]. The analogous protein in mammalian orthoreovirus, σ1, appears to affect tissue tropism and pathogenesis in mice [27]. A hypothesis could be that the sequence diversity between PRV strains (Table 1) may account for the distinct appearances of HSMI and CMS. However, sequencing of the assumed σC/σ1 analogue of PRV from a number of CMS/HSMI outbreaks did not show any distinct pattern (data not shown).

The viral load and localization of totivirus in myocardial cells of heart lesions characteristic of CMS indicate that the totivirus is involved in the disease development. The load of PRV in fish with experimentally induced CMS and the persistence of the infection in the heart indicate that PRV may be opportunistically associated with the development of CMS, but we believe that the totivirus is a more likely causative candidate.

## Competing interests

The authors declare that they have no competing interests.

## Authors' contributions

ML wrote most of the manuscript and did the *in situ *hybridizations. JWN and RF quantified viral loads in tissues and SG participated in the pyrosequencing and performed the sequence comparisons. ABK did all the statistical work and some bioinformatics. JJ, CSP, MM and AJN helped with the sequence comparisons and assembly. CRWN did viral quantification in field samples. TP and ER supervised and guided the project and contributed to the discussion. TT was responsible for the pyrosequencing, viral quantification in field outbreaks, initiation of the project as well as providing funding. All authors read and approved the final manuscript.

## Supplementary Material

Additional file 1 **PRV titres in CMS field outbreaks**. Relative loads of PRV in four different CMS field outbreaks. Click here for file
