# Supplementary material for: A novel totivirus and piscine reovirus (PRV) in Atlantic salmon (Salmo salar) with cardiomyopathy syndrome (CMS)
Source: Virol J. 2010 Nov 10;7:309. doi: 10.1186/1743-422X-7-309 (PMC2994541; doi:10.1186/1743-422X-7-309)
Supplement: Additional file 1 — PRV titres in CMS field outbreaks. Relative loads of PRV in four different CMS field outbreaks. [file 1743-422X-7-309-S1.DOC]

Table S1. Relative loads of PRV in four different CMS field outbreaks.

| **Sample** | **Tissue** | **Pathological changes** | **Relative viral load** |
| --- | --- | --- | --- |
| 440-3 | Heart/kidney | Moderate | 2.3776E-05 |
| 440-4 | Heart/kidney | Severe | 5.6700E-02 |
| 440-5 | Heart/kidney | Severe | 2.3081E-02 |
| 440-6 | Heart/kidney | Moderate | 1.4005E-03 |
| 440-7 | Heart/kidney | Moderate | 4.8401E-03 |
| 440-8 | Heart/kidney | Mild | 9.5946E-05 |
| 479-1 | Kidney | Moderate | 1.0880E-03 |
| 479-2 | Kidney | Severe | 5.7906E-04 |
| 479-3 | Kidney | Severe | 1.8974E-04 |
| 479-4 | Kidney | Moderate | 5.6857E-04 |
| 479-5 | Kidney | Mild | 5.7393E-05 |
| 423-1 | Kidney | Mild | 1.4862E-05 |
| 423-2 | Kidney | Mild | 5.0001E-04 |
| 423-3 | Kidney | Moderate | 1.5563E-04 |
| 423-4 | Kidney | Severe | 3.2941E-04 |
| 423-5 | Kidney | Severe | 1.1732E-02 |
| 222-1 | Heart | Severe | 2.6900E-03 |
| 222-2 | Heart | Severe | 6.4509E-01 |
| 222-3 | Heart | Severe | 1.6053E-02 |
| 222-5 | Heart | Severe | 3.8645E-01 |
| 222-10 | Heart | Severe | 7.4836E-02 |
| Healthy, farmed fish (median value)* | Misc. |  | 2.0686E-05 |

* - data from [14].
